# Effects of Feeding Grapevine Branch–Leaf Silage on Growth Performance, Serum Biochemical Parameters, Rumen Microbial Diversity, and Metabolism in Kazakh Rams

**DOI:** 10.3390/ani16111600

**Published:** 2026-05-24

**Authors:** Kadeliya Abudureyimu, Linhai Song, Buweiaizhaer Maimaitimin, Subinuer Abuduli, Yuxin Zhou, Yongkuo Li, Zhijun Zhang, Wei Shao, Liang Yang, Wanping Ren

**Affiliations:** 1 College of Animal Science, Xinjiang Agricultural University, Urumqi 830052, China; 13699915053@163.com (K.A.); 15009360416@163.com (L.S.); 17590513307@163.com (B.M.); 13649970090@163.com (S.A.); zyxgy1123@163.com (Y.Z.); 13289929530@163.com (Y.L.); dksw@xjau.edu.cn (W.S.); yangliangagu@sina.com (L.Y.); 2 Xinjiang Uygur Autonomous Region Academy of Animal Husbandry Sciences, Urumqi 830011, China; zzj850916@sina.com

**Keywords:** antioxidant indices, grapevine branch and leaf silage, growth performance, immune parameters, Kazakh ram, rumen metabolism

## Abstract

Grapevine branch and leaf silage (GBLS), a polyphenol-rich unconventional forage, exhibits antimicrobial and antioxidant properties that can benefit animal health and productivity. A total of 60 healthy six-month-old Kazakh rams (43.29 ± 4.55 kg, *p* > 0.05 for initial body weight among groups) were randomly assigned to three dietary groups, each consisting of four replicates with five rams per replicate. The control group (CK) was fed a basal diet based on whole-plant corn silage, whereas the experimental groups received diets in which 50% (GBLS50%) or 100% (GBLS100%) of the corn silage was replaced with GBLS. A 10-day adaptation period preceded the 90-day formal feeding trial. Results showed a significant quadratic response for average daily gain (ADG) and average daily feed intake (ADFI) across GBLS substitution rates (*p* < 0.05), with the 50% level yielding the highest values. Specifically, ADFI at the 50% replacement level was significantly higher than that of the control (*p* < 0.05), confirming an inverted U-shaped response with 50% as the optimal substitution rate. However, in-depth analysis of serum biochemical parameters revealed that GBLS supplementation significantly reduced serum concentrations of total cholesterol, triglycerides, urea nitrogen, interleukin-1β (IL-1β), tumor necrosis factor-α (TNF-α), and malondialdehyde (MDA), while significantly increasing levels of immunoglobulins (IgA, IgM, IgG), superoxide dismutase (SOD), and catalase (CAT) (*p* < 0.05). Rumen fermentation analysis showed that the GBLS50% group had significantly lower concentrations of acetate, butyrate, and total volatile fatty acids (VFA) (*p* < 0.05). In the rumen microbiota study, no significant differences were observed in alpha or beta diversity or at the phylum level between groups (*p* > 0.05); however, the abundance of Lactobacillus gasseri was significantly reduced in the GBLS50% group (*p* < 0.05). Metabolomic profiling identified 43 significantly altered metabolites—27 upregulated (e.g., PE (18:1(9Z)/0:0) and 12,14-pentacosadiynoic acid) and 16 downregulated (e.g., deoxyadenosine). Kyoto Encyclopedia of Genes and Genomes (KEGG) pathway enrichment analysis highlighted purine metabolism as a significantly altered pathway (*p* < 0.05), providing insight into the potential metabolic mechanisms underlying the physiological effects of GBLS in rams. In conclusion, replacing 50% of whole-plant corn silage with grapevine branch and leaf silage improves growth performance trends and significantly enhances immunity and antioxidant capacity in Kazakh rams.

## Simple Summary

Grapevine branches and leaves are abundant agricultural by-products in China, especially in Xinjiang. Ensiling these materials offers a promising way to ease forage scarcity and reduce feed costs in sheep farming. In this study, whole-plant corn silage was replaced by grapevine branch–leaf silage at 0%, 50% and 100% on a dry matter basis in diets for Kazakh rams. Compared with the 0% replacement control group, 50% and 100% substitution reduced serum cholesterol, triglycerides, urea nitrogen, inflammatory factors and oxidative stress indicators, while raising immunoglobulin levels and antioxidant enzyme activities. Grapevine branch–leaf silage also enhanced purine metabolism and upregulated key ruminal metabolites. Currently, limited studies have focused on the application of grapevine branch–leaf silage in Kazakh rams. Collectively, these results provide reliable practical references for utilizing grapevine-derived by-products as alternative roughage sources. Notably, the optimal feeding effect was achieved when 50% of whole-plant corn silage was replaced with grapevine branch–leaf silage under the present experimental conditions. This feeding pattern facilitates the development of circular agriculture, cuts down feeding expenses and improves production efficiency, thereby laying a solid scientific foundation for the sustainable exploitation of unconventional feed resources.

## 1. Introduction

In 2022, Xinjiang’s grape output reached 3.1648 million tons, accounting for approximately one-fifth of China’s total grape production and ranking first nationwide [1,2]. Grape cultivation requires extensive pruning, which consequently generates massive agricultural wastes, primarily grapevine branches and leaves [3]. However, the utilization rate of these viticultural by-products remains low, and they are often discarded as horticultural waste. Traditional disposal methods, such as incineration, cause severe resource waste and environmental pollution [4]. Notably, recent studies have indicated that these vine by-products can be used as feed materials, offering an alternative to conventional disposal approaches. Grapevine branches and leaves are rich in polyphenols and other bioactive compounds with antioxidant, antibacterial, and anti-inflammatory properties, which can improve animal health and production performance [5]. In a previous study by Du et al., replacing 40% of corn straw with grapevine branches and leaves significantly improved the average daily gain and feed intake of lambs [6]. Similarly, Xia demonstrated that supplementing diets with varying proportions of grape pomace markedly enhanced the growth performance of meat sheep [7].

Kazakh sheep are classified as one of the three major coarse-wool sheep breeds in China and are defined as a native meat- and fat-purpose sheep breed in Xinjiang [8]. Superior characteristics are exhibited by this breed, including rapid growth rate, high carcass yield, strong adaptability to fibrous diets, and favorable disease resistance [9]. The largest population of Kazakh sheep is distributed in Xinjiang, and this breed possesses great development potential. In preliminary trials, the silage fermentation process of grapevine branches and leaves was optimized through controlled fermentation trials [10]. However, limited research has been conducted on the application of grapevine branch and leaf silage both domestically and internationally, especially regarding its dietary supplementation for meat sheep. Accordingly, Kazakh rams were selected as experimental animals in the present study, and the effects of different substitution ratios of whole-plant corn silage with GBLS were investigated in terms of growth performance, serum biochemical indices, rumen microbial diversity and metabolic profiles. Distinct differences in nutritional components exist between GBLS and whole-plant corn silage. Hence, this study is not designed for simple nutritional substitution; instead, the unconventional feed resource with unique nutritional characteristics is systematically evaluated, and its comprehensive effects on the physiological functions of Kazakh rams are explored after partial replacement of whole-plant corn silage. Large quantities of vine biomass are generated through grape pruning, and the low utilization rate of such biomass brings ecological and economic pressures [4]. The development of GBLS as an innovative feed resource can not only reduce feed costs, improve breeding efficiency and lower reliance on conventional feed ingredients, but also facilitate the value enhancement and high-value utilization of by-products from the viticulture industry.

## 2. Materials and Methods

### 2.1. Animal Ethics Statement

All animal procedures were conducted in strict adherence to the institutional guidelines for animal welfare and ethics, and were granted formal ethical approval by the Experimental Animal Welfare and Ethics Committee of Xinjiang Agricultural University (Approval No. 20240816; dated 29 November 2024).

### 2.2. Experimental Materials

The raw grapevine branch and leaf materials were collected from grape cultivation areas in Turpan City, Xinjiang Uyghur Autonomous Region, China. The GBLS was prepared via 90-day fermentation using a 3% molasses solution supplemented with a mixed microbial feed additive (predominantly containing *Lactococcus lactis* and *Lactobacillus buchneri* at a viable concentration of 1.3 × 10^11^ CFU/g). The microbial inoculant utilized for fermentation was acquired from Hansen Company (Yiyang, Hunan, China). Molasses was obtained from COFCO Tunhe Changji Sugar Industry Co., Ltd. (Changji, China). The weighing scale was supplied by Shanghai Yaohua Weighing Apparatus Co., Ltd. (Shanghai, China). Laboratory consumables, including measuring tapes, blood collection needles, 5 mL blood collection tubes, 2 mL cryovials, and sterile gloves, were all procured from Xinjiang Ziqi Biotechnology Co., Ltd. (Urumqi, China).

### 2.3. Experimental Design and Animal Feeding Management

#### 2.3.1. Animal and Experimental Design

This trial was conducted from November 2024 to February 2025 at a commercial sheep farm in Wugongtai Town, Hutubi County, Changji Hui Autonomous Prefecture, Xinjiang Uyghur Autonomous Region, China, with a total experimental duration of 100 days. A total of 60 healthy male Kazakh sheep at 6 months of age with similar initial body weights (43.29 ± 4.55 kg) were obtained from Manas Xinao Livestock Co., Ltd. (Manas County, Changji City, Changji Hui Autonomous Prefecture, Xinjiang Uyghur Autonomous Region, China). All sheep were randomly allocated to three experimental groups, and no significant differences in initial body weight were observed among groups (*p* > 0.05). Each group was designed with four replicates, and five rams were included in each replicate. The control group (CK) was fed a diet without GBLS, while whole-crop corn silage was substituted with GBLS at proportions of 50% and 100% in the GBLS50 and GBLS100 groups, respectively. A 10-day adaptive feeding period was arranged prior to the 90-day formal feeding trial.

The entire experiment was conducted during winter. The average outdoor temperature fluctuated from −5 °C to −20 °C throughout the trial, while the indoor temperature of the enclosures was steadily maintained at 5–10 °C. Such environmental conditions were consistent with the cold tolerance characteristics and optimal breeding requirements of local Kazakh sheep. The stable indoor feeding environment was utilized to maintain the physiological homeostasis of experimental sheep, which effectively eliminated the confounding interference of cold stress on growth performance, serum biochemical indices, and rumen fermentation parameters. Consequently, reliable experimental data were acquired to accurately evaluate the productive performance of Kazakh sheep under suitable physiological conditions.

#### 2.3.2. Experimental Animal Management

Prior to the commencement of the trial, the sheep housing facility was thoroughly disinfected. Experimental sheep were subjected to deworming treatment via neck subcutaneous injection of ivermectin (0.2 mg/kg), followed by routine gastrointestinal health management before group allocation. All animals were fed at 07:00 and 19:00 daily, and *ad libitum* access to drinking water was provided throughout the experimental period. All additional feeding and environmental management conditions were kept consistent among groups. A total mixed ration (TMR) feeding system was adopted in this experiment, and the experimental diets were formulated in accordance with the Standard for Raising Meat Sheep (NY/T 816-2004) [11]. The dietary composition and nutritional levels of the experimental diets are presented in Table 1, while the nutritional components of whole-crop corn silage and grape branch and leaf silage are compared in Table 2.

### 2.4. Sampling

#### 2.4.1. Growth Performance Data Collection

Body weight measurements were performed at 07:00 on days 0 and 90 of the trial following a 12 h feed deprivation period to ensure uniform fasting conditions. Each sheep was weighed three times, and the averaged values were recorded as the initial body weight (IBW) and final body weight (FBW), respectively. Net weight gain and average daily gain (ADG, g/d) were calculated using the following formula: ADG = (FBW − IBW)/trial period (days). Daily feed provision and residual feed amounts were continuously recorded during the feeding phase. The average daily feed intake (ADFI, g/head/d) was calculated as follows: ADFI = total feed intake (g)/number of days/number of animals.

#### 2.4.2. Blood Sample Collection

Blood samples (10 mL) were collected from the jugular vein of each ram at 6:00 a.m. on days 0, 30, 60, and 90 using sterile vacuum blood collection tubes (containing no anticoagulant; KANGJIAN, Cat# KJ010, Jiangsu Kangjian Medical Supplies Co., Ltd., Taizhou, Jiangsu, China), with consistent timing across all animals to minimize circadian variation. Samples were allowed to clot at room temperature for 30 min, then immediately centrifuged at 3500 r/min for 15 min using a centrifuge (Model GT1, Beili brand, Beijing Beili Centrifuge Co., Ltd., Beijing, China). The resulting serum was transferred into 1.5 mL sterile polypropylene centrifuge tubes (Genview, Cat# GV-MCT-150-C, Beijing Dingguo Changsheng Biotechnology Co., Ltd., Beijing, China) and stored at −20 °C in a freezer (Model DW-25L262, Haier Biomedical, Qingdao, China) until further analysis.

#### 2.4.3. Rumen Fluid Collection

On day 90 of the experiment, 8 experimental sheep with similar body weights were selected from the group with the best growth performance and the CK group. Rumen fluid was collected 3 h after morning feeding, following the method described by Li et al. [13] with minor modifications. Oral intubation was performed using a rumen fluid sampler (Model GCYQ-1-A, Shanghai Yifan Biotechnology Co., Ltd., Shanghai, China) to obtain samples. The initial 50 mL of effluent, which may contain saliva and oral secretions, was discarded, and a subsequent 50 mL of rumen fluid was collected. The sample was immediately filtered through four layers of sterile gauze, and the pH value was determined on-site using a portable pH meter (Model 8601, AZ Instrument Corp., Shanghai, China). Following measurement, the rumen fluid was aliquoted into cryotubes (Genview, Cat# GV-MCT-200-C, Beijing Dingguo Changsheng Biotechnology Co., Ltd., Beijing, China) and rapidly transferred to a liquid nitrogen tank (Model YDS-30, Sichuan Jin Feng Liquid Nitrogen Container Co., Ltd., Chengdu, China) for long-term storage prior to further analysis. All procedures were conducted in strict compliance with the Laboratory Biosafety General Requirements [14].

#### 2.4.4. Preparation and Processing of Grapevine Branch and Leaf Raw Materials for Silage Production

The preparation and hygiene quality control of grapevine branch and leaf silage strictly adhere to the local standard of the Xinjiang Uygur Autonomous Region, “Technical Specification for Feeding Fattening Sheep with Silage-Type Total Mixed Ration Based on Grapevine Branches and Leaves” [15]. Raw materials are sourced from fresh grapevine branches and leaves pruned during the current growing season, exhibiting no visible mold and free from soil contamination. During silage production, the materials are thoroughly chopped using a forage chopper (Model 9Z-6A, Luoyang Sida Agricultural Machinery Co., Ltd., Luoyang, Henan, China), layered with compaction, and stored in airtight silos to ensure a stable anaerobic fermentation environment. Upon opening, silage is removed daily following the principle of “from surface to core, continuous withdrawal” to minimize exposure and maintain feed quality. Prior to feeding, each batch of silage is visually and olfactorily inspected for color and odor; any material displaying mold growth or off-odors is discarded. All procedures are conducted to ensure compliance with the mandatory requirements of the National Standard for Feed Hygiene of the People’s Republic of China [16].

### 2.5. Analysis of Dietary Chemical Composition

The test diet samples were initially dried at 105 °C to constant weight in accordance with the national standard “Determination of Moisture in Feed” [17] to determine dry matter content, using an electric thermostatic drying oven (Model DHG-9240A, Jinghong, Shanghai, China). Subsequently, the dried samples were ground using a laboratory grinder (Model FW-100, Tianjin Taisite Instrument Co., Ltd., Tianjin, China) and passed through a 1 mm sieve for further analysis. The determination of conventional nutritional components strictly followed official Chinese national standard methods: crude protein (CP) was analyzed using the Kjeldahl nitrogen method as specified in GB/T 6432-2018 [18] using a Kjeldahl nitrogen analyzer (Model K1100, Hanon Instruments, Jinan, China); crude fat (EE) was determined according to GB/T 6433-2025 [19] using a Soxhlet extractor (Model SOX-06, Shanghai Hude Scientific Instruments Co., Ltd., Shanghai, China); crude ash (Ash) was measured following the procedure outlined in GB/T 6438-2025 [20] using a muffle furnace (Model SX-4-10, Tianjin Zhonghuan Electric Furnace Co., Ltd., Tianjin, China); calcium (Ca) was quantified in compliance with GB/T 6436-2018 [21] using a spectrophotometer (Model 722N, Shanghai Yidian Analytical Instrument Co., Ltd., Shanghai, China); and total phosphorus (P) was assessed using the spectrophotometric method described in GB/T 6437-2018 [22] using the same spectrophotometer (Model 722N, Shanghai Yidian Analytical Instrument Co., Ltd., Shanghai, China). Neutral detergent fiber (NDF) and acid detergent fiber (ADF) contents were determined using the detergent fiber analysis method of Van Soest et al. [23].

### 2.6. Serum Sample Analysis

The serum biochemical parameters, including total protein, total cholesterol, and triglycerides, were analyzed at Beijing Huaying Biotechnology Research Institute. These analytes were quantified using commercially available assay kits (Beijing Zhongying Biotechnology Research Institute Co., Ltd., Beijing, China; Cat# ZY-001) via colorimetric methods on a fully automated biochemical analyzer (Model BS-420, Mindray Bio-Medical Electronics Co., Ltd., Shenzhen, Guangdong, China). For cytokine markers such as interleukin-1β (IL-1β) and tumor necrosis factor-α (TNF-α), a commercially available enzyme-linked immunosorbent assay (ELISA) kit (Beijing Huaying Biotechnology Research Institute, Beijing, China; Cat# HY-IL1β-001) was used for quantitative determination with an ELISA microplate reader (Model DR-200BS, Huawei Delang, Wuxi, Jiangsu, China). The assay procedures were conducted strictly in accordance with the manufacturer’s instructions: serum samples were added to microplates pre-coated with specific capture antibodies and subjected to incubation and washing steps. Enzyme-conjugated detection antibodies were then added, followed by incubation and a subsequent wash cycle. A chromogenic substrate solution was introduced to initiate the colorimetric reaction, which was terminated by the addition of stop solution. Absorbance values were measured at 450 nm, and the concentrations of target cytokines were calculated based on a standard curve derived from reference standards.

### 2.7. Analysis of Rumen Fermentation Parameters

The pH of the filtered rumen fluid was measured immediately using a portable pH meter (Model 8601, AZ Instrument Corp., Shanghai, China). The concentrations of volatile fatty acids (VFA) were quantified via gas chromatography–mass spectrometry (GC–MS) using a gas chromatograph-mass spectrometer (Model 7890B-5977B, Agilent Technologies, Inc., Santa Clara, CA, USA) at the Feed Quality and Safety Evaluation Center, Institute of Feed Research, Xinjiang Academy of Animal Science (Urumqi, China).

### 2.8. Analysis of Rumen Microbial Diversity

The microbial diversity in rumen fluid samples was analyzed by Hangzhou Jingsen Biotechnology Co., Ltd. (Hangzhou, China) using high-throughput sequencing targeting the bacterial 16S rRNA gene. PCR amplification was performed with primers specific to the V3–V4 hypervariable regions of the 16S rRNA gene, using the region-specific forward primer 338F (5′-barcode + ACTCCTACGGGAGGCAGCA-3′) and reverse primer 806R (5′-GGACTACHVGGGTWTCTAAT-3′) on a thermal cycler (Veriti 96-well Thermal Cycler, Applied Biosystems, Carlsbad, CA, USA). The thermal cycling protocol included an initial denaturation at 98 °C for 30 s, followed by 25–27 cycles of denaturation at 98 °C for 15 s, annealing at 50 °C for 30 s, and extension at 72 °C for 30 s, with a final extension step at 72 °C for 5 min. Amplified products were purified prior to library construction, which involved sequential steps of end repair, 3′-end adenylation, and ligation of indexed adapters. BECKMAN AMPure XP beads (Cat# A63881, Beckman Coulter, Brea, CA, USA) were used for purification after each step. Library quality was assessed using LabChip (LabChip GX, PerkinElmer, Waltham, MA, USA), and paired-end sequencing (2 × 250 bp) was conducted on the Illumina NovaSeq 6000 platform (Illumina, Inc., San Diego, CA, USA) with the NovaSeq 6000 SP Reagent Kit (500 cycles, Cat# 20027465, Illumina, Inc., San Diego, CA, USA).

Microbial community composition and diversity were analyzed based on the resulting 16S rRNA gene sequencing data. Alpha diversity indices were calculated using QIIME2 (version 2023.7) and visualized as box plots in R (version 4.2.0). Beta diversity was evaluated through principal coordinate analysis (PCoA), computed and displayed as two-dimensional scatter plots in the R environment. Relative abundances of microbial taxa at the phylum and genus levels were determined using QIIME2 and presented as bar plots via the ggplot2 package (version 3.4.3) in R. Venn diagrams depicting shared and unique operational taxonomic units (OTUs) across experimental groups were generated using the ggvenn package (version 0.1.10) in R.

### 2.9. Rumen Metabolomics Analysis

Rumen fluid metabolomic analysis was conducted at Hangzhou Jingsen Biotechnology Co., Ltd. The profiling procedure was performed as follows: samples were thoroughly vortexed using a vortex mixer (MX-S, Scilogex, Rocky Hill, CT, USA), and 100 μL aliquots were mixed with 400 μL of cold methanol. After vortexing for 1 min, the mixtures were subjected to five cycles of ultrasonication in an ice-water bath (1 min per cycle) using an ultrasonic cleaner (KQ-500E, Kunshan Ultrasonic Instruments Co., Kunshan, China), followed by a 1 min pause between each cycle. The extracts were then centrifuged at 14,000 rpm and 4 °C for 10 min using a centrifuge (Model H2050R, Xiangyi, Shanghai, China). Supernatants were carefully collected, dried overnight under vacuum using a vacuum concentrator (Concentrator plus, Eppendorf, Hamburg, Germany), and reconstituted in 80 μL of 50% acetonitrile aqueous solution.

The resulting solutions were transferred to autosampler vials (Agilent, Cat# 5182-0717, Agilent Technologies, Santa Clara, CA, USA) and analyzed by liquid chromatography–mass spectrometry (LC–MS) using an LC-MS system (Vanquish UHPLC coupled with Q-Exactive Orbitrap, Thermo Fisher Scientific, Waltham, MA, USA).

To identify metabolic differences between experimental groups, orthogonal partial least squares discriminant analysis (OPLS-DA) was first applied to construct multivariate models and generate score plots for visualization using SIMCA software (version 14.1, Sartorius, Sweden). Significant differential metabolites were selected based on a combination of criteria: variable importance in projection (VIP) values from the OPLS-DA model, *p*-values derived from univariate statistical tests, and fold changes. Subsequently, differential metabolites were annotated against the Kyoto Encyclopedia of Genes and Genomes (KEGG) database to map associated metabolic pathways. Pathway enrichment analysis was performed using the MetPA database and the hypergeometric test algorithm to identify statistically significant metabolic pathways.

### 2.10. Data Analysis

Experimental data were preliminarily processed using Excel 2010 (Microsoft Corp., Redmond, WA, USA). One-way ANOVA was performed using SPSS 27.0 (IBM Corp., Armonk, NY, USA), followed by Duncan’s multiple range test for post hoc comparisons. Given that the substitution levels (0%, 50%, 100%) were equally spaced, orthogonal polynomial contrasts were additionally conducted to test linear and quadratic trends. For rumen fermentation parameters, independent samples *t*-test was employed. The results are presented as mean and its standard error (SEM). Statistical significance was defined at *p* < 0.05 and non-significance at *p* > 0.05. Statistical analyses were conducted according to standard biostatistical principles [24] using IBM SPSS Statistics 27.0 [25].

## 3. Results

### 3.1. Effects of Feeding Different Proportions of Grapevine Branch and Leaf Silage on Growth Performance of Kazakh Rams

As shown in Table 3, the net weight gain and average daily gain of the GBLS 50% group were 15.3% higher than those of the GBLS 100% group (*p* < 0.05). However, no significant differences were observed in net weight gain or ADG between the GBLS 50% and CK groups (*p* > 0.05). The ADFI in the GBLS 50% group was 14.1% higher than that in the CK group (*p* < 0.05), whereas no significant difference in ADFI was detected between the GBLS 50% and GBLS 100% groups (*p* > 0.05). Notably, orthogonal polynomial contrasts demonstrated a significant quadratic response for ADG and ADFI across increasing substitution rates (0%, 50%, 100%) (*p* < 0.05).

### 3.2. Effects of Feeding Different Proportions of Grapevine Branch and Leaf Silage on Serum Biochemical, Immune and Antioxidant Indexes of Kazakh Rams

#### 3.2.1. Effects of Feeding Different Proportions of Grapevine Branch and Leaf Silage on Serum Biochemical Parameters in Kazakh Rams

As shown in Table 4, the total cholesterol (TC) concentration in the GBLS 100% group was significantly lower than that in the CK group at 30, 60, and 90 days (*p* < 0.05). However, no significant differences were observed between the GBLS 100% and GBLS 50% groups at any of these time points (*p* > 0.05). At 90 days, the triglyceride (TG) concentration in the GBLS 100% group was 21.2% lower than that in the CK group (*p* < 0.01), whereas no significant difference was detected between the GBLS 100% and GBLS 50% groups (*p* > 0.05). TG at 90 days exhibited a significant linear decreasing trend across increasing GBLS substitution rates (*p* < 0.01). At 30 days, the blood urea nitrogen (BUN) concentration in the GBLS 100% group was 15.9% lower than that in the CK group (*p* < 0.05), while no significant difference was observed between the GBLS 100% and GBLS 50% groups (*p* > 0.05). No significant differences were found in the concentrations of total protein (TP) among all groups at any time point throughout the experimental period (*p* > 0.05).

#### 3.2.2. Effects of Feeding Different Proportions of Grape Shoot and Leaf Silage on Immune Factor of Kazakh Rams

As shown in Table 5, at 30 and 90 days, the immunoglobulin A (IgA) content in both the GBLS 50% and GBLS 100% groups was significantly higher than that in the CK group (*p* < 0.05). At 60 days, IgA levels in the GBLS 50% and GBLS 100% groups were 19% and 27% higher, respectively, than in the CK group (*p* < 0.01), with no significant difference between the two experimental groups (*p* > 0.05). At 30, 60, and 90 days, IgA levels showed a significant linear increasing trend across substitution rates (*p* < 0.05). At 30 and 60 days, immunoglobulin G (IgG) content in the GBLS 100% group was significantly higher than in the CK group (*p* < 0.05), while no significant difference was observed between the GBLS 100% and GBLS 50% groups (*p* > 0.05). IgG also showed a significant linear increasing trend at 30 and 60 days (*p* < 0.05). At 30 days, immunoglobulin M (IgM) content in the GBLS 50% and GBLS 100% groups was 24.1% and 34.5% higher, respectively, than in the CK group (*p* < 0.01), and no significant differences were found between the two experimental groups (*p* > 0.05). IgM exhibited a significant linear increasing trend at 30 days (*p* < 0.01).

#### 3.2.3. Effects of Feeding Different Proportions of Grapevine Branch and Leaf Silage on Antioxidant Indexes and Inflammatory Factors in Kazakh Rams

As shown in Table 6, at 60 days, the IL-1β level in the CK group was significantly higher than that in the GBLS 100% group (*p* < 0.05), but showed no significant difference compared to the GBLS 50% group (*p* > 0.05). At 90 days, the IL-1β level in the CK group was 43.8% higher than that in the GBLS 100% group (*p* < 0.01) and 17.7% higher than that in the GBLS 50% group (*p* < 0.05), while the IL-1β level in the GBLS 50% group was 22.1% higher than that in the GBLS 100% group (*p* < 0.05). At 30, 60, and 90 days, IL-1β levels showed a significant linear decreasing trend across substitution rates (*p* < 0.05). At 30, 60, and 90 days, the TNF-α level and malondialdehyde (MDA) concentration in the CK group were significantly higher than those in the GBLS 100% group (*p* < 0.05), with no significant differences observed when compared to the GBLS 50% group (*p* > 0.05). TNF-α and MDA also showed significant linear decreasing trends at 30, 60, and 90 days *(p* < 0.05). At 30 and 90 days, the superoxide dismutase (SOD) concentration in the GBLS 100% group was significantly higher than that in both the CK group and the GBLS 50% group (*p* < 0.05), whereas the SOD concentration in the GBLS 50% group was higher than in the CK group, though not significantly (*p* > 0.05). SOD exhibited a significant linear increasing trend at 30, 60, and 90 days (*p* < 0.05). At 90 days, catalase (CAT) concentrations in the GBLS 50% and GBLS 100% groups were 17.64% and 25.9% higher, respectively, than in the CK group (*p* < 0.01), with no significant difference between the two experimental groups (*p* > 0.05). CAT showed a significant linear increasing trend at 90 days (*p* < 0.01).

### 3.3. Effects of Feeding Different Proportions of Grapevine Branch and Leaf Silage on Rumen Fermentation and Rumen Microbial Diversity of Kazakh Rams

The above results indicate that the growth performance of Kazakh rams in the GBLS 50% group was the highest. Therefore, the CK group and the GBLS 50% group were selected for 16S rRNA gene sequencing to analyze the rumen microbial community composition. In the subsequent results, group A refers to the GBLS 50% group and group B refers to the CK group.

#### 3.3.1. Effects of Feeding Different Proportions of Grapevine Branch and Leaf Silage on Rumen Fermentation of Kazakh Rams

As shown in Table 7, the acetic acid (AA) concentration in the GBLS 50% group was 19.8% lower than that in the CK group (*p* < 0.05); the butyric acid (BA) concentration in the GBLS 50% group was 46.4% lower than that in the CK group (*p* < 0.01). The total volatile fatty acids (TVFA) concentration in the GBLS 50% group was 21.8% lower than that in the CK group (*p* < 0.05), whereas no significant differences were observed between the two groups in terms of pH value, propionic acid (PA), isobutyric acid (IBA), isovaleric acid (IVA), valproic acid (VA), or their concentrations (*p* > 0.05).

#### 3.3.2. Analysis of Alpha and Beta Diversity of Rumen Microorganisms

**Figure 1:**
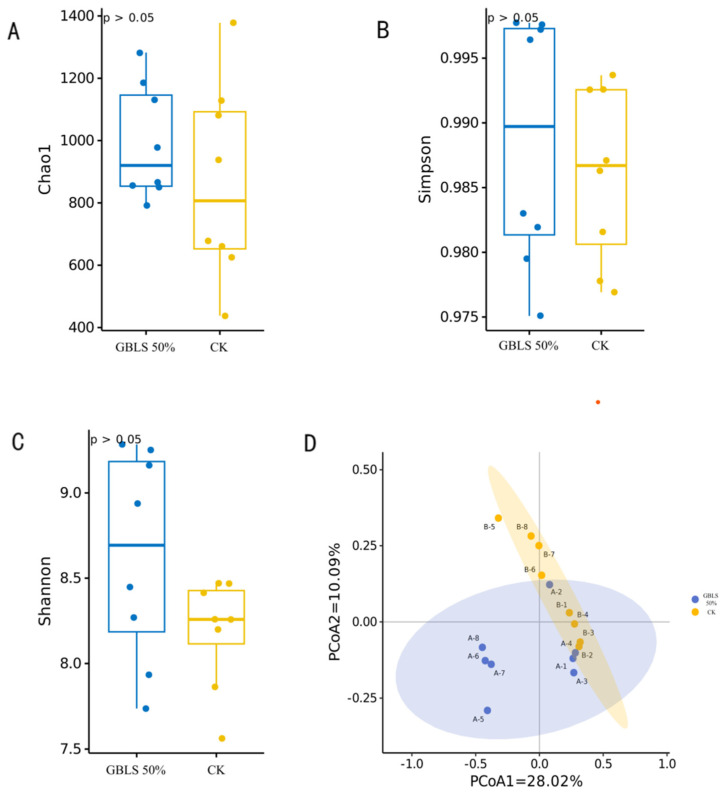
Analysis of rumen microbial α- and β-diversity between the GBLS 50% group and the CK group in Kazakh rams. (**A**) Chao1 index; (**B**) Simpson index; (**C**) Shannon index; (**D**) Principal coordinate analysis (PCoA) plot based on Bray–Curtis distances (PCoA1 = 28.02%, PCoA2 = 10.09%).

Figure 1 presents the α-diversity and β-diversity indices of rumen microorganisms in Kazakh rams from the GBLS 50% and CK groups. As shown in Figure 1A–C, there were no significant differences in the Chao1, Shannon, and Simpson indices between the GBLS 50% and CK groups (*p* > 0.05). As shown in Figure 1D, the microbial communities exhibited low intergroup variability between the GBLS 50% and CK groups, with no significant difference in β-diversity (*p* > 0.05).

#### 3.3.3. Composition of Rumen Microbiota at the Phylum Level

**Figure 2:**
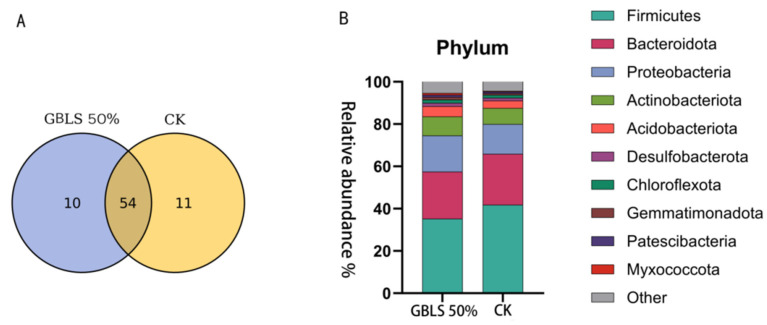
Phylum-Level Rumen Microbial Community Structure in the GBLS 50% Group versus the CK group of Kazakh Rams. (**A**) represents shows the Venn diagram; (**B**) represents displays the phylum-level abundance distribution in the GBLS 50% group versus the CK group.

As shown in Figure 2A, at the phylum level, the GBLS 50% and CK groups shared 54 core microbial OTUs. The GBLS 50% group contained 10 unique OTUs, while the CK group had 11 unique OTUs. As shown in Figure 2B, *Firmicutes*, *Bacteroidota*, and *Proteobacteria* were identified as the dominant phyla in both the GBLS 50% and CK groups. The relative abundances of *Firmicutes* and *Bacteroidota* in the GBLS 50% group were 6.58% and 1.85% lower, respectively, than those in the CK group.

#### 3.3.4. Composition of Rumen Microbiota at the Genus Level

**Figure 3:**
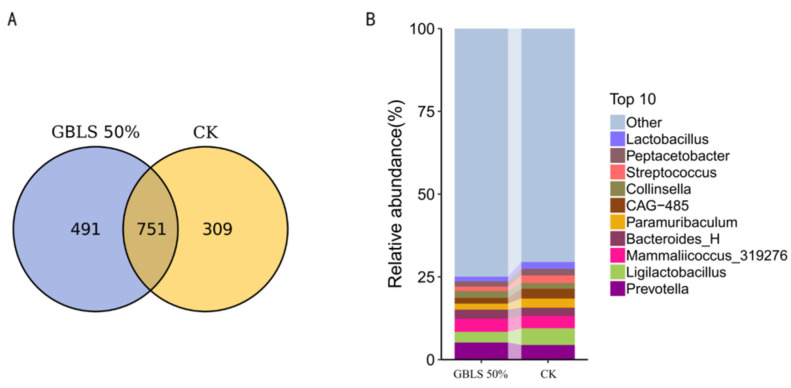
Genus-Level Rumen Microbial Community Structure in the GBLS 50% Group versus the CK group of Kazakh Rams. (**A**) represents shows the Venn diagram; (**B**) represents displays the genus-level abundance distribution in the GBLS 50% group versus the CK group.

As shown in Figure 3A, at the genus level, the GBLS 50% and CK groups shared 751 core microbial OTUs. The GBLS 50% group contained 491 unique OTUs, while the CK group had 309 unique OTUs. As shown in Figure 3B, *Prevotella* and *Ligilactobacillus* were identified as the dominant genera in both the GBLS 50% and CK groups. The relative abundance of *Ligilactobacillus* in the GBLS 50% group was significantly lower than that in the CK group (*p* < 0.05).

#### 3.3.5. LEfSe Analysis

**Figure 4:**
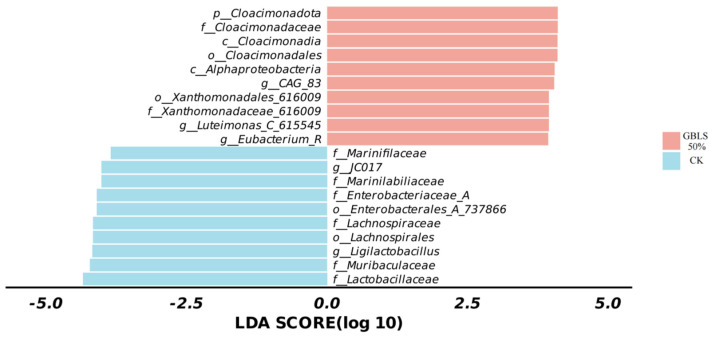
LEfSe analysis diagram of Rumen Microbiota in the GBLS 50% Group versus the CK group of Kazakh Rams.

As shown in Figure 4, the phylum p_*Cloacimonadota* was significantly enriched in the GBLS 50% group. At the genus level, g_*CAG*_83, g_*Luteimonas_C_615545*, and g_*Eubacterium_R* were significantly enriched in the GBLS 50% group, whereas g_*JC017* and g_*Ligilactobacillus* were significantly enriched in the CK group.

### 3.4. Effects of Feeding Different Proportions of Grapevine Branch and Leaf Silage on Rumen Metabolomics of Kazakh Rams

#### 3.4.1. Metabolite OPLS-DA Results

**Figure 5:**
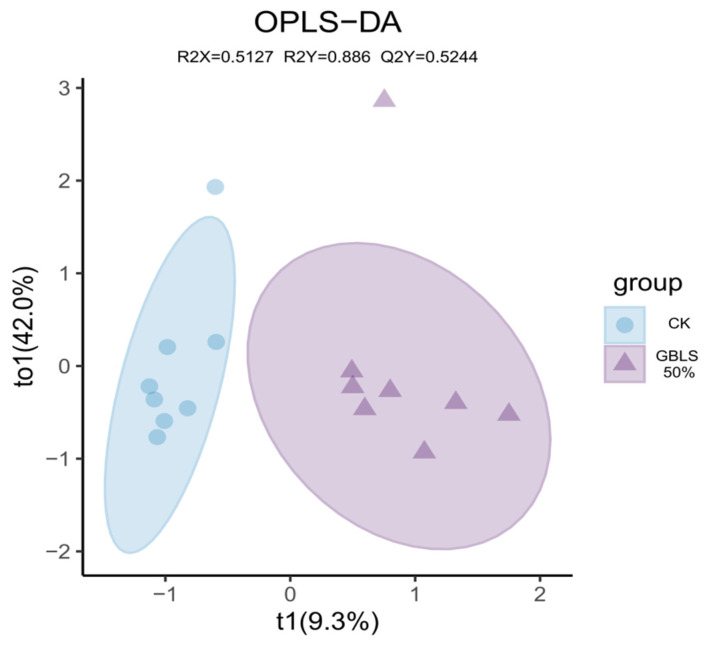
OPLS-DA Score Plot of the GBLS 50% Group versus the CK group. “Group” indicates the experimental grouping. The horizontal axis represents the predictive principal component, where the direction indicates intergroup differences; the vertical axis represents the orthogonal principal component, where the direction reflects intragroup variations; the percentage indicates the explained variance of the component of the dataset. Each point in the figure represents an individual sample. Samples in the same group are represented by the same color.

As shown in Figure 5, the first principal coordinate (t1) of the rumen metabolic samples accounted for 9.3% of the variation in rumen metabolism, while the second principal coordinate (tO1) accounted for 42.0%. In this study, samples within each group exhibited clear clustering.

#### 3.4.2. Differential Metabolite Analysis

**Figure 6:**
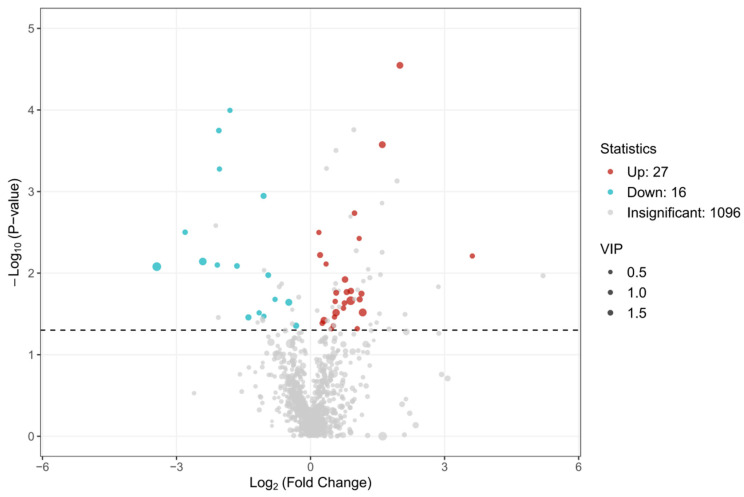
Volcano Plot of Differential Metabolites in the GBLS 50% Group versus the CK group.

Volcano plots were used to analyze differential metabolites in rumen fluid. As shown in Figure 6, a total of 1096 differential metabolites—detected in both positive and negative ion modes—were identified, encompassing both significant and non-significant features. Based on the screening criteria of fold change (FC) > 1, VIP > 1.0, and *p* < 0.05, a total of 43 significantly differential metabolites were identified, including 27 up-regulated and 16 down-regulated metabolites.

**Figure 7:**
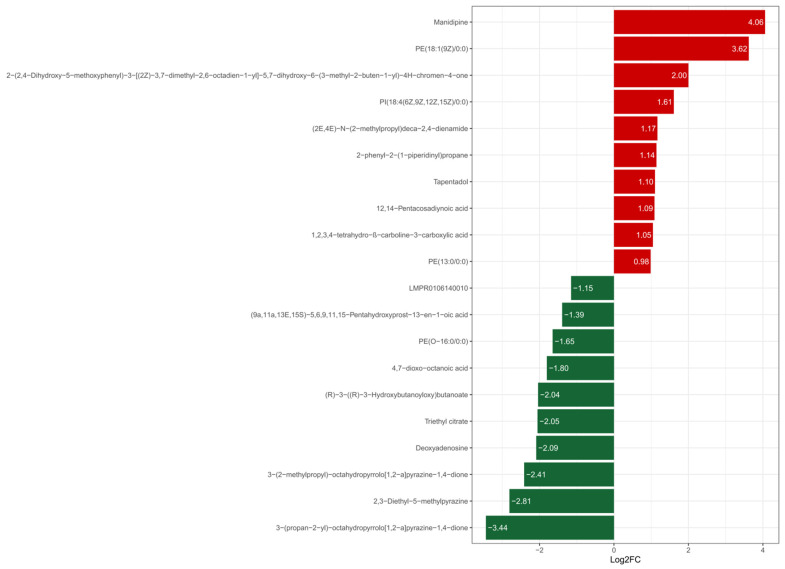
Bar Chart of Fold Change in the GBLS 50% Group vs. Red dots denote up-regulated metabolites, while green dots indicate down-regulated metabolites.

As shown in Figure 7, lysophospholipid-class differential metabolites such as PE(18:1(9Z)/0:0), PI(18:4(6Z,8Z,12Z,15Z)/0:0), and PE(13:0/0:0), as well as plant antibacterial substance-class metabolites including 2-(2,4-Dihydroxy-5-methoxyphenyl)-3-[(2Z)-3,7-dimethyl-2,6-octadien-1-yl]-5,7-dihydroxy-8-(3-methyl-2-buten-1-yl)-4H-chromen-4-one and 12,14-Pentacosadiynoic acid, were significantly up-regulated in the GBLS 50% group (*p* < 0.05). In contrast, metabolites such as Deoxyadenosine and Triethyl citrate were significantly down-regulated (*p* < 0.05).

#### 3.4.3. KEGG Enrichment Analysis Results of Differential Metabolites

**Figure 8:**
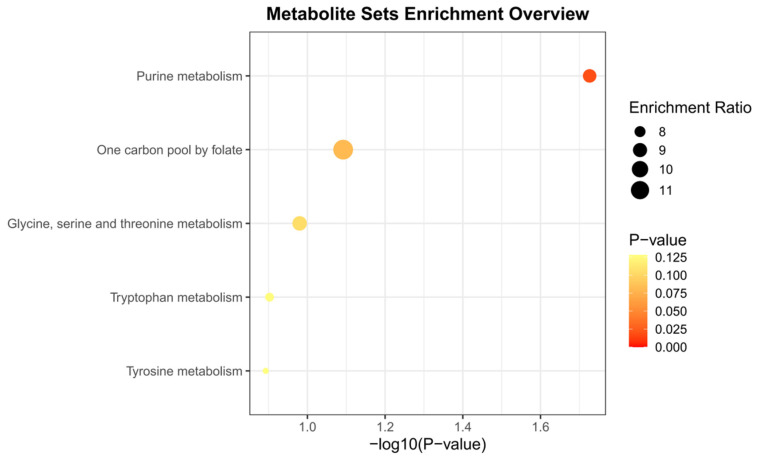
Bubble Plot of Impact Factors for Differential Metabolites in the GBLS 50% Group versus the CK group. The CK group the volcano plot displays the distribution of differential metabolites, with the *x*-axis representing the log_2_ (fold change) and the *y*-axis showing the −log_10_ (*p*-value).

As shown in Figure 8, KEGG enrichment analysis of differential metabolites showed that the differential metabolites in rumen fluid between the two groups were primarily enriched in five metabolic pathways: purine metabolism, one-carbon pool by folate, glycine, serine and threonine metabolism, tryptophan metabolism, and tyrosine metabolism. Among these, the purine metabolism pathway was significantly enriched (*p* < 0.05).

## 4. Discussion

### 4.1. Effects of Feeding Different Proportions of Grapevine Branch and Leaf Silage on Growth Performance of Kazakh Rams

Our previous research on GBLS demonstrated that it is rich in bioactive compounds such as resveratrol (unpublished data) and characterized by high crude protein content (Table 2). During the ensiling process, a substantial amount of lactic acid is produced as a metabolic byproduct of lactic acid bacterial proliferation, which plays a crucial role in promoting efficient fermentation [26]. The addition of molasses further enhanced the acidity and aromatic profile of the silage, resulting in a pleasant odor similar to distiller’s grains or pickles and thereby improving feed palatability. In this trial, orthogonal polynomial contrast analysis was performed for these equally spaced substitution gradients. The results showed a significant quadratic response for average daily gain (ADG) and average daily feed intake (ADFI) across GBLS substitution rates, with the 50% substitution level yielding the highest values. Specifically, ADFI at the 50% level was significantly higher than that in the control group, indicating an obvious inverted U-shaped dose-dependent response; thus, 50% was identified as the optimal substitution dosage. The growth-promoting potential of grapevine by-products has been validated in previous ruminant feeding trials. Du et al. [6] reported that replacing corn stover with 40% grapevine branches increased lamb ADG and average daily feed intake by 13.04% and 7.24%, respectively. Similarly, incorporating grapevine branches to replace sorghum stover also improved sheep growth performance [27], and appropriate supplementation of 10–15% grape pomace effectively elevated lamb ADG [28]. Collectively, these studies confirm that moderate inclusion of grapevine-derived forage positively regulates ruminant growth. Nevertheless, the present gradient experiment revealed a noticeable decline in ADG when corn silage was completely replaced by GBLS. This growth inhibition can be logically explained by the reduction in dietary starch concentration following full substitution. As the primary energy substrate for ruminants, insufficient starch intake inevitably constrains nutrient deposition and weight gain. In comparison, the optimal growth outcome observed in the 50% GBLS group was closely associated with its higher voluntary feed intake, which provided sufficient nutrients for somatic growth. Based on the combined gradient responses and growth trajectories, the 50% substitution level represented the optimal dosage for balancing dietary energy supply and bioactive substance intake in Kazakh rams.

### 4.2. Effects of Feeding Different Proportions of Grapevine Branch and Leaf Silage on Serum Biochemical, Immune and Antioxidant Indexes of Kazakh Rams

#### 4.2.1. Effects of Feeding Different Proportions of Grapevine Branch and Leaf Silage on Serum Biochemical Parameters in Kazakh Rams

Blood TC and TG concentrations are regarded as pivotal biomarkers for evaluating animal lipid metabolism, and a negative correlation is established between these indicators and fat utilization efficiency [29]. In the current gradient feeding trial, although slight linear declining trends for serum TC and TG were observed with increasing GBLS inclusion, these linear responses were not statistically significant. Nevertheless, obvious differences in serum biochemical parameters were detected among individual treatments at the same sampling time points. The TC concentrations in the 100% GBLS group were significantly decreased throughout the feeding period (days 30, 60, and 90), and an extremely lower TG concentration was observed on day 90 when compared with the control group. Serum urea nitrogen (BUN), which is widely accepted as a critical indicator of protein metabolism and nitrogen utilization efficiency [30], was also markedly reduced in the 100% GBLS group on day 30. These metabolic variations were primarily attributed to the abundant bioactive compounds present in GBLS. The lipid-regulating potential of bioactive compounds derived from grapevine by-products has been validated in numerous previous studies. Lipid synthesis-related enzymes can be suppressed by flavonoids extracted from grapevine branches, thereby reducing serum triglyceride levels [31]. Serum cholesterol accumulation can also be alleviated by polyphenolic compounds in grape processing by-products [32]. Furthermore, serum BUN concentrations have been significantly decreased in beef cattle fed grape seed proanthocyanidins, indicating an improvement in nitrogen deposition efficiency [33]. Accordingly, the decreased serum TC, TG, and BUN concentrations observed in GBLS-treated rams in the present study indicated an improvement in lipid and nitrogen utilization, which was mediated by the synergistic effects of flavonoids, polyphenols, and proanthocyanidins contained in GBLS. Nevertheless, relatively lower starch content and nutrient density in GBLS compared with corn silage were also considered partial causes for these altered serum biochemical indicators.

#### 4.2.2. Effects of Feeding Different Proportions of Grapevine Branch and Leaf Silage on Immune and Biochemical Indices of Kazakh Rams

The serum concentration of immunoglobulins is regarded as a critical parameter for objectively reflecting the immune capacity of animals, and an important quantitative indicator is provided for the evaluation of humoral immune status [34]. In the present gradient feeding trial, a significant linear increase in serum immunoglobulin levels was observed with the elevation of GBLS substitution rates. Nevertheless, obvious differences in immune parameters were also detected among individual treatments at identical sampling time points. On day 30, higher IgA and IgM concentrations were detected in all GBLS-supplemented groups, while a significant elevation in IgG was uniquely identified in the 100% GBLS group. Consistently higher immunoglobulin contents were sustained in GBLS-containing groups throughout the feeding period, with the maximal immune enhancement effect recorded in the 100% substitution group. Consistent with the findings of the present trial, improvements in immune function have been documented in beef cattle fed diets supplemented with grape pomace, and enhanced disease resistance has been reported in sheep offered grapevine pellet feed [35,36]. The immunomodulatory capacity of GBLS is intrinsically attributed to its abundant phytochemical compounds. Considerable amounts of tannins (1.19–1.27%) and resveratrol (0.01–0.11%) have been quantified in grapevine branches [37]. Polyphenols and flavonoids derived from grape materials are capable of modulating lymphocyte proliferation and immunoglobulin secretion, thereby exerting potent regulatory effects on humoral immunity [38]. Accordingly, the dose-dependent elevation of serum immunoglobulins in GBLS-fed Kazakh rams can be explained by the synergistic biological activities of endogenous polyphenols and flavonoids contained in grapevine branches and leaves.

#### 4.2.3. Effects of Feeding Different Proportions of Grapevine Branch and Leaf Silage on Antioxidant Indexes and Inflammatory Factors in Kazakh Rams

Tumor necrosis factor-α (TNF-α) is defined as a typical pro-inflammatory cytokine that is involved in the regulation of systemic inflammatory responses [39], while malondialdehyde (MDA) is regarded as a critical marker for evaluating lipid peroxidation and oxidative damage in organisms [40]. Superoxide dismutase (SOD) is recognized as an essential antioxidant enzyme by which excess reactive oxygen species are eliminated, oxidative stress is alleviated, and cellular damage is prevented to achieve anti-inflammatory effects [41,42]. In the present gradient feeding trial, a significant linear reduction in serum IL-1β, TNF-α, and MDA concentrations was observed with the elevation of GBLS substitution rates. Meanwhile, a significant linear elevation in antioxidant enzyme activities (SOD and CAT) was recorded as the GBLS dosage increased. Nevertheless, obvious differences in these antioxidant and inflammatory parameters were also detected among individual treatments at identical sampling time points. Lower pro-inflammatory cytokines and MDA contents, as well as markedly higher antioxidant enzyme activities, were observed in GBLS-supplemented groups relative to the control group. The antioxidant and anti-inflammatory capacities of GBLS are intrinsically attributed to its abundant secondary metabolites. Proanthocyanidins enriched in grapevine branches have been verified to alleviate inflammatory responses through the suppression of pro-inflammatory cytokine secretion [43,44]. Enhanced antioxidant enzyme activities and reduced MDA accumulation have also been documented in multiple animal models following proanthocyanidin supplementation [45,46]. Furthermore, the expression of antioxidant enzymes can be upregulated by quercetin and other flavonoid derivatives contained in grape extracts, thereby strengthening systemic antioxidant defense capacity [47]. Accordingly, inflammatory reactions are suppressed and redox status is optimized through the synergistic effects of intrinsic polyphenols, anthocyanins, and flavonoids in GBLS, which contributes to the improvement of the general health status of Kazakh rams. It should be clarified that although the cited literature primarily focused on grape seeds and pomace, these by-products share similar bioactive polyphenolic compounds with GBLS; such compositional similarity provides reliable mechanistic references. However, this similarity in specific bioactive substances cannot be regarded as nutritional equivalence or interchangeability among grape seeds, grape pomace, and grapevine branch and leaf silage with respect to overall nutrient composition, fiber structure, digestibility, or metabolic effects.

### 4.3. Effects of Feeding Different Proportions of Grapevine Branch and Leaf Silage on Rumen Fermentation and Rumen Microbial Diversity of Kazakh Rams

Given the superior comprehensive performance of the 50% GBLS substitution group in terms of growth traits and serum metabolic profiles, we further compared rumen fermentation parameters and microbial communities between the control and 50% GBLS groups. Rumen pH acts as a critical indicator for evaluating rumen environmental stability. The results showed that rumen pH values in both groups remained within a normal physiological range (7.10–7.12), indicating that partial replacement of corn silage with 50% GBLS did not disrupt rumen pH homeostasis in Kazakh rams. Volatile fatty acids (VFA), including acetate, propionate, and butyrate, serve as the primary energy substrates for ruminants [48]. The 50% GBLS group exhibited lower concentrations of acetate, butyrate, and total VFA relative to the control group.

Ruminal VFA production is strongly correlated with dietary starch content. High-starch corn silage promotes the proliferation of starch-degrading bacteria and accelerates VFA accumulation [49]. Partial substitution of corn silage with GBLS reduced dietary starch levels, thereby decreasing fermentable substrates and lowering ruminal VFA concentrations. This variation in fermentation characteristics coincided with the decreased serum BUN level, collectively indicating that GBLS contains less rapidly available energy than corn silage. Notably, rumen pH remained stable across all treatments, confirming that GBLS can be safely used as a mild, non-acidogenic roughage for sheep feeding.

Rumen microorganisms are essential for ruminal digestion, and their community composition profoundly affects nutrient metabolism and immune function while maintaining rumen homeostasis [50]. No significant differences in alpha diversity indices (Chao1, Shannon, and Simpson) were observed between the two groups, suggesting that moderate GBLS substitution did not alter the overall richness and diversity of rumen microorganisms. At the phylum level, *Firmicutes* and *Bacteroidota* dominated the rumen bacterial community. *Firmicutes* primarily degrades fibrous substances to provide energy for the host, whereas *Bacteroidota* efficiently decomposes complex carbohydrates [51,52]. Although no significant intergroup differences were detected, the relative abundances of these two dominant phyla slightly declined in the GBLS group, which was likely attributed to the reduced dietary starch proportion.

*Prevotella* plays a vital role in cellulose and protein degradation [53]. Consistent with previous studies, higher dietary crude protein elevates the abundance of *Prevotella* [54]. Accordingly, the 50% GBLS group had a greater relative abundance of *Prevotella,* which may be explained by the slight increase in dietary crude protein after corn silage was partially replaced with GBLS. *Ligilactobacillus* is a newly defined genus of Gram-positive, non-spore-forming bacteria previously classified within the *Lactobacillus salivarius* group [55]. These bacteria produce lactic acid and other organic acids during glucose fermentation. Excessive proliferation of lactobacilli causes lactic acid accumulation, decreases ruminal pH, and increases the risk of subacute ruminal acidosis [56]. In the present trial, the relative abundance of *Ligilactobacillus* decreased in the 50% GBLS group. This alteration may be attributed to the inhibitory effects of GBLS-derived polyphenols (e.g., tannins and gallic acid) on Gram-positive bacteria [57]. GBLS supplementation suppresses the proliferation of *Ligilactobacillus,* thereby reducing lactic acid accumulation and mitigating ruminal acidosis risk. Collectively, although most bacterial genera remained unchanged across treatments, the distinct reduction in *Ligilactobacillus* abundance was deemed a beneficial alteration for maintaining rumen microecological stability.

### 4.4. Effects of Feeding Different Proportions of Grapevine Branch and Leaf Silage on Rumen Metabolomics of Kazakh Rams

#### 4.4.1. Effects of Feeding Different Proportions of Grapevine Branch and Leaf Silage on Differential Metabolites in the Rumen of Kazakh Rams

Dietary modification can lead to alterations in rumen-derived metabolites. In this experiment, 43 metabolites were significantly different between the GBLS 50% and CK groups, with 27 upregulated and 16 downregulated. Notably, metabolites such as PE (18:1(9Z)/0:0) and 12,14-pentacosadiynoic acid were significantly upregulated, whereas deoxyadenosine was significantly downregulated. PE (18:1(9Z)/0:0) is a naturally occurring lysophospholipid and an analog of plasmenyl lysophosphatidylethanolamine (LPE). As a lysophospholipid, high hydrophilicity is exhibited by LPE [58]. It has been demonstrated that the utilization of fibrous dietary components can be enhanced and milk production and growth performance can be improved by dietary supplementation with lysophospholipids in dairy cows [59]. Similarly, significant increases in the digestibility of dry matter, organic matter, and crude protein have been documented in lambs fed lysophospholipid supplementation, thereby facilitating the improvement of growth performance [60]. In the present study, higher ADG and ADF values were recorded in the 50% GBLS group relative to the CK group. These findings indicated that fiber utilization capacity may be enhanced by the upregulation of PE (18:1(9Z)/0:0) under GBLS treatment, thereby promoting feed intake and weight gain in Kazakh rams. Furthermore, key metabolites associated with lipid metabolism (e.g., PE (18:1(9Z)/0:0)) were upregulated in the rumen of Kazakh rams fed the 50% GBLS diet. Concurrently, decreased serum TC and TG concentrations were detected in the GBLS group, which reflected the optimized lipid utilization efficiency. The improved lipid metabolic capacity was regarded as one of the critical factors responsible for the superior daily weight gain of rams in the 50% GBLS treatment.

12,14-Pentacosadiynoic acid is categorized as a straight-chain fatty acid derivative, while the screened isoprenyl flavonoid in the present study represents a structurally complex flavonoid compound. The metabolic regulatory potential of isoprenyl flavonoids has been validated in previous animal trials. In a broiler feeding experiment, elevated cecal short-chain fatty acid concentrations and improved nutrient metabolism were detected after dietary supplementation with isoprenyl flavonoid-rich Epimedium [61], and reduced methane production and enhanced feed efficiency were further verified via in vitro ruminal fermentation assays [62]. In the present trial, a slightly higher ADG value and a decreased abundance of *Ligilactobacillus* were recorded in the 50% GBLS group. The synergistic upregulation of 12,14-pentacosadiynoic acid and isoprenyl flavonoids was considered responsible for the inhibition of acid-producing bacteria. Furthermore, comparable antibacterial properties are exhibited by these two metabolites, and the proliferation of Gram-positive bacteria can be suppressed [63]. Collectively, the optimized microbial composition alleviated ruminal acidosis risk, improved feed utilization efficiency, and thereby contributed to the superior growth performance of Kazakh rams.

(2E,4E)-N-(2-methylpropyl) deca-2,4-dienamide (pellitorine) is classified as a fatty acid amide-type plant-derived alkaloid [64]. The biological activity of pellitorine is determined by its conjugated diene system and amide functional group. The regulatory effects of this alkaloid on lipid metabolism and immune-inflammatory responses have been documented in animal trials [65]. The activation of the nuclear factor kappa B (NF-κB) signaling pathway can be inhibited by pellitorine, thereby reducing the secretion of pro-inflammatory cytokines (e.g., TNF-α and IL-6) [66,67,68]. In the present trial, significant upregulation of pellitorine was detected in the 50% GBLS group, and decreased concentrations of circulating pro-inflammatory cytokines were synchronously observed. This metabolic variation provided a reasonable molecular explanation for the lower serum TNF-α and IL-1β levels in GBLS-fed rams. Accordingly, the ameliorated inflammatory status in the 50% GBLS group was attributed to the elevated abundance of pellitorine.

Deoxyadenosine is a direct precursor of dATP (deoxyadenosine triphosphate), and decreased deoxyadenosine abundance reflects reduced dATP synthesis. Excessively accumulated dATP has been verified to inhibit ribonucleotide reductase (RNR), block the biosynthesis of other deoxyribonucleoside triphosphates (dNTPs), disrupt DNA replication progression, and induce cytotoxicity [69]. Furthermore, dATP accumulation can trigger excessive assembly of the lymphocyte apoptosome (Apaf-1/caspase complex), thereby facilitating lymphocyte apoptosis and promoting the secretion of pro-inflammatory mediators [70,71]. In the present trial, the downregulated deoxyadenosine level was accompanied by attenuated inflammatory responses and improved immune performance in the 50% GBLS group. This metabolic alteration provides a reasonable molecular explanation for the elevated serum IgA and IgM concentrations as well as the decreased IL-1β level in GBLS-fed rams. GBLS supplementation may effectively restrain abnormal dATP accumulation and its downstream cytotoxic effects by suppressing the conversion of deoxyadenosine to dATP. Consequently, pathological lymphocyte apoptosis and excessive pro-inflammatory cytokine release are alleviated, which further enhances systemic anti-inflammatory capacity and immune function. This metabolic regulation is considered the core molecular basis for the improved immune performance of the 50% GBLS group. Collectively, the synergistic modulation of pellitorine and deoxyadenosine is considered to underlie the optimized anti-inflammatory and immune responses in GBLS-fed Kazakh rams.

#### 4.4.2. KEGG Enrichment Analysis of Differential Rumen Metabolites in Kazak Rams Fed with Different Proportions of Grapevine Branch and Leaf Silage

KEGG enrichment analysis is defined as a bioinformatics approach based on the KEGG pathway database, which is employed to screen biologically meaningful metabolic pathways and clarify the intrinsic correlations between metabolites and phenotypic characteristics [72]. In the present trial, purine metabolism was identified as the most significantly altered pathway among the ruminal differential metabolites between the 50% GBLS and CK groups. Purines and pyrimidines are regarded as critical nitrogen sources for microbial nitrogen transformation and utilization in the rumen, and approximately 20% of microbial nitrogen has been verified to be derived from purine and pyrimidine metabolic processes [73]. Significant enrichment of purine and pyrimidine metabolism has previously been detected in weaned Hu sheep supplemented with herbal additives, and the improved growth performance was speculated to be associated with dietary bioactive compounds such as flavonoids and alkaloids [74]. Purine metabolism constitutes the core subsection of nucleotide metabolism, and purine nucleotides are indispensable for microbial proliferation and cellular metabolic activities [75]. The growth performance and antioxidant capacity of dairy calves have been significantly improved by exogenous nucleotide supplementation [76]. Similarly, enhanced feed intake, weight gain, and rumen fermentation capacity have been documented in nucleotide-fed Holstein calves [77]. In the present study, significant enrichment of purine metabolism was synchronously observed with superior growth performance in the 50% GBLS group. This metabolic alteration was attributed to GBLS-derived alkaloids and flavonoids, which may modulate the activity of microbial nucleotide synthetase. Consequently, the de novo synthesis and recycling of purine nucleotides were promoted, and rumen nitrogen utilization efficiency was further optimized. Collectively, the enhanced cellular metabolic activity and antioxidant defense system are considered to underlie the improved production performance of GBLS-fed rams. A potential causal relationship between purine metabolism and growth performance is therefore inferred, although further mechanistic verification is still required. The present findings provide novel insights into improving nutritional efficiency in ruminants through dietary regulation of rumen microbial metabolic networks. Further integrated analyses combining metagenomics and metabolomics should be conducted to elucidate the underlying molecular mechanisms and to screen key functional microorganisms.

In addition to purine metabolism, tryptophan metabolism was identified as another critical functional pathway closely associated with host immune regulation. Tryptophan is an essential amino acid involved in protein synthesis, and its regulatory effects on physiological metabolism, gastrointestinal microecology, and immune homeostasis have been widely documented [78]. Although not statistically significant, a notable enrichment trend of the tryptophan metabolism pathway was observed in the 50% GBLS group. This metabolic tendency was highly consistent with the elevated serum IgA and IgG concentrations in GBLS-fed Kazakh rams. Tryptophan and its downstream metabolites can modulate immunoglobulin secretion and lymphocyte proliferation, thereby maintaining intestinal immune homeostasis and regulating systemic immune responses [79]. Together with the improved humoral immune parameters observed in the 50% GBLS group, this enrichment in tryptophan metabolism is suggested to contribute to the optimized immune status. The abundant polyphenols and alkaloids in GBLS may facilitate the absorption and immunometabolic conversion of tryptophan, consequently improving protein digestibility and host immune capacity. Nevertheless, the precise molecular regulatory mechanisms underlying this non-significant metabolic trend warrant further investigation.

## 5. Conclusions

In the present trial, replacing 50% of whole-plant corn silage with grapevine branch and leaf silage effectively improved feed intake, immune status, and antioxidant capacity in Kazakh rams. Rumen microbial diversity analysis revealed that dietary supplementation with grapevine branch and leaf silage did not substantially alter the ruminal alpha diversity or the relative abundance of dominant microbial taxa at the phylum level. Additionally, rumen metabolomic profiling identified significant changes in key differential metabolites following the dietary substitution: metabolites including PE (18:1 (9Z)/0:0), 12,14-pentacosadiynoic acid, and (2E,4E)-N-(2-methylpropyl) deca-2,4-dienamide were markedly upregulated, while deoxyadenosine was significantly downregulated. KEGG enrichment analysis further indicated that purine metabolism was the most prominently enriched metabolic pathway. This optimized feeding strategy enables cost-effective and high-efficiency sheep production and facilitates the development of sustainable, eco-friendly animal husbandry. Collectively, these findings provide insights into improving the growth performance and metabolic health of rams, and establish a feasible technical model for the development of green agriculture and circular economy from both production and ecological perspectives. For future research directions, long-term feeding trials involving multiple growth stages, breeding patterns, and livestock species are required. Furthermore, multi-omics integrated analysis should be adopted to systematically elucidate the potential molecular mechanisms and causal relationships underlying the immunometabolic responses induced by grapevine branch and leaf silage.

## Figures and Tables

**Table 1:** Ingredient composition and measured nutrient levels of experimental diets for Kazakh rams (%, dry matter basis).

Item	Treatment ^(1)^		
	CK Group	GBLS 50 Group	GBLS 100 Group
Dietary composition,%			
Whole-plant corn silage	30	15	0
Grapevine branch and leaf silage	0	15	30
Cotton Residue	30	30	30
Corn	24	24	24
Cottonseed Meal	5	5	5
Soybean Meal	5	5	5
Wheat Bran	3.9	3.9	3.9
Premix ^(2)^	1.5	1.5	1.5
Salt	0.3	0.3	0.3
Soda	0.3	0.3	0.3
Total	100	100	100
Nutritional level ^(3)^			
Metabolizable Energy (ME) (MJ/kg)	10.89	10.75	10.60
Crude protein (CP) (%)	13.00	13.46	13.33
Crude fat (EE) (%)	4.24	4.32	4.26
Neutral Detergent Fiber (NDF) (%)	65.23	64.65	65.36
Acid Detergent Fiber (ADF) (%)	25.36	25.65	25.61
Crude ash (Ash) (%)	8.95	9.12	9.20
Calcium Ca (%)	0.75	0.79	0.73
Phosphorus P (%)	0.39	0.38	0.39

^(1)^ CK group: Group fed with whole-plant corn silage; GBLS50% group: Replace the whole-plant corn silage with 50% grapevine branch and leaf silage; GBLS100% group: Replace the whole-plant corn silage with 100% grapevine branch and leaf silage. ^(2)^ Premix composition (per kg): Vitamin A ≥ 80,000 IU, Vitamin D_3_ ≥ 20,000 IU, Vitamin E ≥ 500 IU, Iron (as Ferrous Sulfate) ≥ 1100 mg, Copper (as Tribasic Copper Chloride) 300–600 mg, Zinc (as Zinc Sulfate) 1000–2500 mg, Manganese (as Manganese Sulfate) ≥ 200 mg, Selenium (as Sodium Selenite) 10 mg, Iodine (as Calcium Iodate) ≥ 10 mg, Calcium 12–18%, Total Phosphorus ≥ 3%, Sodium Chloride 16–20%. ^(3)^ All nutritional levels are measured values.

**Table 2:** Comparison of nutritional composition between whole-plant corn silage and grapevine branch and leaf silage (on a dry matter basis).

Whole-Plant Corn Silage ^(1)^		Grapevine Branch and Leaf Silage ^(2)^	
CP (%)	6.43	CP (%)	12.70
EE (%)	3.22	EE (%)	4.64
NDF (%)	44.5	NDF (%)	45.14
ADF (%)	26.8	ADF (%)	24.69

^(1)^ The nutritional composition data of whole-plant corn silage were obtained from He et al. (2026) [12]. ^(2)^ All nutrient indicators for grapevine branch and leaf silage are based on experimental measurements.

**Table 3:** Effects of Feeding Different Proportions of Grapevine Branch and Leaf Silage on the Growth Performance of Kazakh Rams.

Items	Group			SEM	*p*-Value		
	CK Group	GBLS 50 Group	GBLS 100 Group		Group	Linear	Quadratic
IBW/kg	44.5	42.1	43.3	1.03	0.69	0.66	0.46
FBW/kg	61.5	59.4	58.3	1.58	0.77	0.48	0.90
Net Weight Gain/kg	17.0 ^ab^	17.3 ^a^	15.0 ^b^	0.26	0.04	0.35	0.13
ADG (g/d)	188.3 ^ab^	192.3 ^a^	166.8 ^b^	0.15	0.03	0.29	0.04
ADFI/kg	2.13 ^b^	2.43 ^a^	2.28 ^ab^	0.08	0.04	0.43	0.02

CK group: Group fed with whole-plant corn silage; GBLS50% group: Replace the whole-plant corn silage with 50% grapevine branch and leaf silage; GBLS100% group: Replace the whole-plant corn silage with 100% grapevine branch and leaf silage. Different lowercase letters indicate significant differences (*p* < 0.05), and the same letters or no letters indicate no significant differences (*p* > 0.05). IBW: Initial body weight, FBW: Final body weight, NWG: Net Weight Gain, ADG: Average Daily Gain, ADFI: Average Daily Feed Intake.

**Table 4:** Effects of Feeding Different Proportions of Grapevine Branch and Leaf Silage on Serum Biochemical Parameters in Kazakh Rams.

Items	Time	Group			SEM	*p*-Value		
		CK Group	GBLS 50 Group	GBLS 100 Group		Group	Linear	Quadratic
TP (g/L)	0 d	63.1	59.3	61.5	0.95	0.27	0.48	0.15
	30 d	65.1	65.8	66.5	0.51	0.55	0.11	0.29
	60 d	65.5	67.1	65.0	0.63	0.41	0.73	0.20
	90 d	65.8	67.0	65.9	1.02	0.87	0.97	0.60
TG (mmol/L)	0 d	0.21	0.22	0.21	0.01	0.29	0.40	0.19
	30 d	0.24	0.23	0.23	0.01	0.99	0.89	0.96
	60 d	0.26	0.24	0.24	0.01	0.75	0.54	0.68
	90 d	0.33 ^A^	0.30 ^AB^	0.26 ^B^	0.01	<0.01	<0.01	0.92
TC (mmol/L)	0 d	1.20	1.25	1.21	0.02	0.53	0.71	0.29
	30 d	1.49 ^a^	1.43 ^ab^	1.33 ^b^	0.05	0.04	0.13	0.63
	60 d	1.71 ^a^	1.61 ^ab^	1.55 ^b^	0.05	0.02	0.07	0.41
	90 d	1.83 ^a^	1.76 ^ab^	1.63 ^b^	0.08	0.04	0.16	0.56
BUN (mg/dL)	0 d	16.0	15.2	14.9	0.56	0.75	0.47	0.84
	30 d	15.2 ^a^	13.7 ^ab^	12.8 ^b^	0.53	0.02	0.06	0.75
	60 d	15.4	14.6	14.4	0.24	0.25	0.12	0.58
	90 d	15.4	15.1	13.9	0.36	0.19	0.09	0.51

Different uppercase letters among peer data indicate extremely significant differences (*p* < 0.01), different lowercase letters indicate significant differences (*p* < 0.05), and the same letters or no letters indicate no significant differences (*p* > 0.05). TP: Protein, TC: Total Cholesterol, TG: Triglyceride, BUN: Blood Urea Nitrogen.

**Table 5:** Effects of Feeding Different Proportions of Grapevine Branch and Leaf Silage on Immune Factor of Kazakh Rams.

Items	Time	Group			SEM	*p*-Value		
		CK Group	GBLS 50% Group	GBLS 100% Group		Group	Linear	Quadratic
IgA (g/L)	0 d	0.90	0.98	0.94	0.60	0.62	0.80	0.51
	30 d	0.94 ^b^	1.12 ^a^	1.19 ^a^	0.04	0.02	0.01	0.40
	60 d	1.00 ^B^	1.19 ^A^	1.27 ^A^	0.05	<0.01	<0.01	0.41
	90 d	1.06 ^b^	1.20 ^a^	1.34 ^a^	0.04	0.03	0.02	0.58
IgG (g/L)	0 d	15.7	16.7	15.9	0.57	0.73	0.44	0.96
	30 d	16.1 ^b^	18.5 ^ab^	20.8 ^a^	0.79	0.03	0.01	1.00
	60 d	16.5 ^b^	17.1 ^ab^	19.2 ^a^	0.43	0.04	0.04	0.51
	90 d	17.4	18.7	19.0	0.55	0.52	0.29	0.68
IgM (g/L)	0 d	0.58	0.58	0.61	0.02	0.87	0.65	0.81
	30 d	0.58 ^B^	0.72 ^A^	0.78 ^A^	0.03	<0.01	<0.01	0.43
	60 d	0.64	0.60	0.72	0.02	0.13	0.19	0.11
	90 d	0.67	0.65	0.72	0.02	0.50	0.38	0.45

Different uppercase letters among peer data indicate extremely significant differences (*p* < 0.01), different lowercase letters indicate significant differences (*p* < 0.05), and the same letters or no letters indicate no significant differences (*p* > 0.05). IgA: Immunoglobulin A, IgG: Immunoglobulin G, IgM: Immunoglobulin M.

**Table 6:** Effects of Feeding Different Proportions of Grapevine Branch and Leaf Silage on Antioxidant Indices and Inflammatory Factors in Kazakh Rams.

Items	Time	Group			SEM	*p*-Value		
		CK Group	GBLS 50% Group	GBLS 100% Group		Group	Linear	Quadratic
IL-1β (pg/mL)	0 d	31.2	29.1	29.1	1.26	0.74	0.51	0.70
	30 d	25.4	23.6	21.5	0.71	0.07	0.04	0.92
	60 d	26.9 ^a^	24.2 ^ab^	18.8 ^b^	1.34	0.03	0.01	0.57
	90 d	24.6 ^a^	20.9 ^b^	17.1 ^c^	0.91	0.01	<0.01	0.97
TNF-α (pg/mL)	0 d	62.6	58.5	57.6	2.81	0.77	0.50	0.81
	30 d	62.2 ^a^	52.2 ^ab^	44.0 ^b^	2.78	0.02	<0.01	0.85
	60 d	60.9 ^a^	49.7 ^ab^	41.6 ^b^	3.02	0.02	0.01	0.78
	90 d	59.5 ^a^	48.8 ^ab^	39.7 ^b^	2.93	0.01	<0.01	0.88
MDA (nmol/mL)	0 d	4.94	4.70	4.80	0.16	0.84	0.73	0.64
	30 d	4.38 ^a^	3.87 ^ab^	3.24 ^b^	0.18	0.02	<0.01	0.83
	60 d	4.08 ^a^	3.44 ^ab^	2.89 ^b^	0.19	0.02	0.01	0.89
	90 d	3.71 ^a^	3.28 ^ab^	2.69 ^b^	0.16	0.02	0.01	0.76
SOD (U/mL)	0 d	56.5	56.6	58.8	3.52	0.96	0.81	0.90
	30 d	69.3 ^b^	71.2 ^b^	86.1 ^a^	3.40	0.03	0.02	0.34
	60 d	66.1 ^b^	71.0 ^ab^	83.4 ^a^	2.39	0.02	0.02	0.51
	90 d	71.7 ^b^	77.6 ^b^	90.2 ^a^	2.77	0.01	<0.01	0.48
CAT (U/mL)	0 d	30.4	33.5	31.5	0.91	0.41	0.65	0.21
	30 d	31.9	32.9	34.7	1.25	0.69	0.40	0.87
	60 d	31.2	34.3	37.8	1.44	0.18	0.07	0.96
	90 d	31.9 ^B^	37.5 ^A^	40.2 ^A^	1.21	<0.01	<0.01	0.46

Different uppercase letters among peer data indicate extremely significant differences (*p* < 0.01), different lowercase letters indicate significant differences (*p* < 0.05), and the same letters or no letters indicate no significant differences (*p* > 0.05). IL-1β: Interleukin-1 beta, TNF-α: Tumor Necrosis Factor-alpha, MDA: Malondialdehyde, SOD: Superoxide Dismutase, CAT: Catalase.

**Table 7:** Effects of Feeding Different Proportions of Grapevine Branch and Leaf Silage on Rumen Fermentation of Kazakh Rams.

Items	Group		SEM	*p*-Value
	CK Group	GBLS 50% Group		
pH	7.10	7.12	0.02	0.26
AA	137.9 ^a^	110.7 ^b^	0.47	0.04
PA	23.3	20.0	0.58	0.35
IBA	1.17	1.47	0.07	0.12
BA	29.2 ^A^	15.6 ^B^	0.11	<0.01
IVA	1.23	1.61	0.14	0.09
VA	6.25	6.31	0.34	0.27
AA/PA	5.92	5.53	0.65	0.31
TVFA	199.1 ^a^	155.7 ^b^	0.75	0.04

Different uppercase letters among peer data indicate extremely significant differences (*p* < 0.01), different lowercase letters indicate significant differences (*p* < 0.05), and the same letters or no letters indicate no significant differences (*p* > 0.05). AA: Acetic acid, PA: Propionic acid, IBA: Is butyric acid, BA: butyric acid, IVA: Isovaleric acid, VA: Valproic acid, AA/PA: Ratio of acetic acid to propionic acid, TVFA: Total volatile fatty acids.

## Data Availability

The data presented in this study are available on request from the corresponding author.

## Abbreviations

The following abbreviations are used in this manuscript:

Abbreviation	Full term
AA	Acetic acid
ADF	Acid detergent fiber
ADFI	Average daily feed intake
ADG	Average daily gain
Apaf-1	apoptotic protease activating factor-1
BA	Butyric acid
BUN	Blood urea nitrogen
CAT	Catalase
CK	Control group
CP	Crude protein
caspase	cysteine-aspartic proteases
dATP	deoxyadenosine triphosphate
DM	Dry matter
ELISA	Enzyme-linked immunosorbent assay
FBW	Final body weight
GBLS50%	50% grapevine branch–leaf silage group
GBLS100%	100% grapevine branch–leaf silage group
GC-MS	Gas chromatography–mass spectrometry
GLU	Glucose
GSE	Grape seed extract
HDL	High-density lipoprotein
IBW	Initial body weight
IgA	Immunoglobulin A
IgG	Immunoglobulin G
IgM	Immunoglobulin M
IL-1β	Interleukin-1 beta
IL-6	interleukin-6
IBA	Isobutyric acid
IVA	Isovaleric acid
KEGG	Kyoto Encyclopedia of Genes and Genomes
LC–MS	Liquid chromatography–mass spectrometry
LPE	lysophosphatidylethanolamine
MDA	Malondialdehyde
NDF	Neutral detergent fiber
NF-κB	Nuclear factor kappa B
OPLS-DA	Orthogonal partial least squares discriminant analysis
OTU	Operational taxonomic unit
PA	Propionic acid
PCoA	Principal coordinate analysis
RNR	Ribonucleotide reductase
SD	Standard deviation
SOD	Superoxide dismutase
TC	Total cholesterol
TG	Triglyceride
TNF-α	Tumor necrosis factor-alpha
TP	Total protein
TVFA	Total volatile fatty acids
VA	Valeric acid
VFA	Volatile fatty acid(s)
VIP	Variable importance in projection
